# A Comprehensive Retrospective Institutional Study for Decoding Oral Squamous Cell Carcinoma

**DOI:** 10.7759/cureus.54001

**Published:** 2024-02-11

**Authors:** Prathiba Reichal, Roland Prethipa

**Affiliations:** 1 Oral Medicine and Radiology, Saveetha Dental College and Hospitals, Saveetha Institute of Medical and Technical Sciences (SIMATS) Saveetha University, Chennai, IND

**Keywords:** health, quality of life, prevalence, oral squamous cell carcinoma, demography

## Abstract

Background

Oral cancer is found to be the thirteenth most common cancer as stated by the WHO (World Health Organization 2023). Oral squamous cell carcinoma (OSCC) is associated with deleterious oral habits such as smoking, chewing tobacco and betel quid, alcohol consumption, low socioeconomic status, sharp teeth, and various causative factors.

Materials and methods

A three-year retrospective analysis (March 2020-September 2023) was carried out with the available patient records in the Dental Information Archival Software (DIAS) used in a private dental college in Chennai. The demographic data such as age, gender, and habit duration and clinicopathological data such as the anatomical site; tumor, node, and metastasis (TNM) staging; perineural invasion (PNI); lymphovascular invasion (LVI); and oral health-related quality of life were retrieved. Statistical analysis was done using IBM SPSS Statistics for Windows, Version 23.0 (Released 2015; IBM Corp., Armonk, New York, United States).

Results

Males (78.35%) more commonly reported OSCC than females (21.62%), and the majority of them were in the age category of fifth to seventh decades of life. The most affected region was the buccal mucosa with 33.3%, followed by the lower alveolus with 30.63%. The duration of harmful habits varied from one year to more than 40 years, and the majority of the patients had T4a staging (40.54%), followed by T2 staging (29.73%) with a habit duration of more than five years. Approximately 22.52% and 0.9% had PNI and LVI, respectively. The correlation between the two variables was evaluated using the Pearson correlation test and was found to be statistically significant (p < 0.05), i.e., habit to gender and staging with gender were p = 0.027 and p = 0.028, respectively.

Conclusion

The majority of cases reported were found to be at T4a tumor staging with a habitual duration of more than five years, and more than half of the study population had severe compromise in their quality of life. The presence of perineural invasion and lymphovascular invasion has an impact on nodal metastasis, treatment choices, recurrence, and oral health-related quality of life. To address this challenge, oral health programs can implement comprehensive antitobacco counseling strategies, oral cancer public awareness programs to tackle the rising incidence of OSCC, and early oral precancer screening measures to enhance the prevention and overall quality of life of individuals with oral cancer.

## Introduction

The contemporary world is currently advancing toward numerous types of noncommunicable diseases, and cancer is one such disease that leads to death and crippling of life. Oral cancer has been considered a chief public health problem in developing countries, especially India, where it ranks in the third position among the other world countries [1]. Around the world, an approximation of more than 10 million of the population are being affected by cancer, and among them, more than six million deaths are recorded each year [2]. The incidence rate of OSCC (oral squamous cell carcinoma) varies based on its geographic location and has been tagged as the most important cause of mortality and morbidity worldwide. Among all types of cancer, oral cancer is found to be the 13th most common cancer, as stated by the WHO (World Health Organization) in 2023 [3]. In the Indian subcontinent, oral cancers are most prevalent, and 50% of all carcinomas are OSCC, which is most common among the male population and the third most common cancer among the female population [4]. A soaring number of cases of oral squamous cell carcinoma are recorded among elderly females as well as young females [5]. As far as India is concerned, regardless of the presence of various diagnostic and therapeutic advances, the overall incidence and mortality rate associated with oral cancer is still rising gradually, amounting to 6.6 per one lakh. Among them, 3.1 per one lakh of the population is male, and 2.9 per one lakh is female, respectively [6].

The main anatomic sites associated with oral squamous cell carcinoma are the tongue, buccal mucosa, alveolus, floor of the mouth, palate, and a few other sites within the oral cavity. The affected site also varies among the population based on their geographic locations, as noted earlier. Oral squamous cell carcinoma is associated with deleterious oral habits and consumption of alcohol, along with smoking tobacco, that can synergistically cause cancer of the following regions: oral cavity, pharynx, larynx, and esophagus [7]. The rate of smoking in developed countries is in a declining phase due to the effects of numerous public health policies. But regardless of the habit, younger females are affected by oral carcinoma, irrespective of the country being developed or in the developing stage. Still, the root cause of this issue is unknown. Previous studies state that there is a 15-fold increase in the risk of oral squamous cell carcinoma when patients are subjected to a combination of alcohol consumption and tobacco smoking [8]. A former retrospective analysis done by DeAngelis et al. reported that elderly nonsmoking females who do not consume alcohol regularly had significantly worse survival rates, and these findings were novel and authentic [9]. The International Agency for Research on Cancer has confirmed that the use of smokeless tobacco can also be an aggravating factor for oral cancers [10]. Schantz et al. stated that variations in oral cancer can be based on geographical location, age, gender, race, and anatomic site [11]. A preponderance population of oral squamous cell carcinoma-affected patients is diagnosed at stages III or IV, which markedly decreases the survival rate of these patients and causes substantial deterioration of the patient’s quality of life [5]. The purpose of the present study was to shed light on the epidemiological profiles such as demographics, anatomical site, clinicopathological patterns, and oral health-related quality of life of oral squamous cell carcinoma patients reported in a private dental institute, in Chennai.

## Materials and methods

Inclusion and exclusion criteria

This retrospective study included 111 OSCC patients’ data retrieved from patient records in the Dental Information Archival Software (DIAS) used in Saveetha Dental College and Hospitals, Saveetha Institute of Medical and Technical Sciences (SIMATS), Chennai, between March 2020 and September 2023 with a purposive sampling technique and ethical approval (IHEC/SDC/UG-1871/23/OMR/332) obtained from the same institute to conduct the study and extract the data. Clinicopathological information of each case, such as age (categorized into seven groups: 21-30 years, 31-40 years, 41-50 years, 51-60 years, 61-70 years, 71-80 years, and 81-90 years), gender, habit duration, anatomical region (buccal mucosa, tongue, maxillary alveolus, mandibular alveolus, combination of maxillary and mandibular alveolus, and floor of the mouth), TNM (tumor, nodal, and metastasis) staging 8th edition, 2017 at the time of diagnosis [12], perineural invasion, lymphovascular invasion, and overall oral health were assessed with a modified Oral Health Impact Profile (OHIP) questionnaire comprising of seven questions on difficulty in swallowing, taste, mastication, speech, and pain or discomfort in the oral cavity of the patient that were categorized under five domains [13]. The questions were asked by the physician to the patient postoperatively, and the scores were recorded based on the responses obtained from the patient, which ranged between 0 and 10, to evaluate the oral health-related quality of life. A total of 156 cases were retrieved from the DIAS system, of which 45 cases were excluded due to histological uncertainty and irregular follow-up data for the OSCC cases.

**Figure 1:**
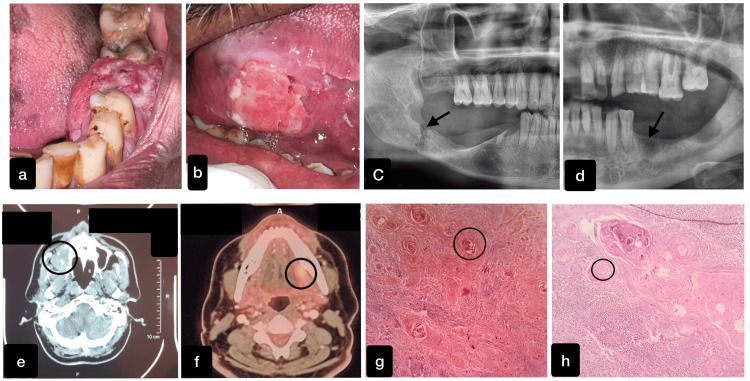
Clinical, radiological, and histopathological images of OSCC. a and b represent the ulceroproliferative growth of the oral squamous cell carcinoma (OSCC) present on the mandibular alveolus and the lateral border of the tongue, respectively. c and d denote the orthopantomogram showing the pathological fracture and osteolytic changes with invasive borders by OSCC in the right and left mandibular region. e shows the axial section of the computed tomography depicting the destruction of the right side walls of the maxillary sinus. f represents the positron emission tomography (PET) depicting focal hypermetabolism seen as an enhancing lesion in the left lateral border of the posterior one-third of the tongue. g and h depict the presence of keratin pearls present on the histological section that is appreciated by a special stain Ayoub-Shklar.

The collected data was entered in MS Excel (Microsoft Corporation, Redmond, Washington, United States) and analyzed statistically with IBM SPSS Statistics for Windows, Version 23.0 (Released 2015; IBM Corp., Armonk, New York, United States). The collected data were subjected to a thorough statistical analysis, which produced the final results. Below are some oral squamous cell carcinoma cases reported to Saveetha Dental College and Hospitals, SIMATS, Chennai. The normality of the data was checked using the Kolmogorov-Smirnov test. The significance level is taken at a p-value of <0.05 with a confidence interval of 95%. The Pearson correlation test was used to evaluate the link between the two variables and provide information about the strength and direction of the correlation. Figure 1 represents few of the OSCC patients clinical, radiological, and histopathological images retrieved from DIAS. 

## Results

**Figure 2:**
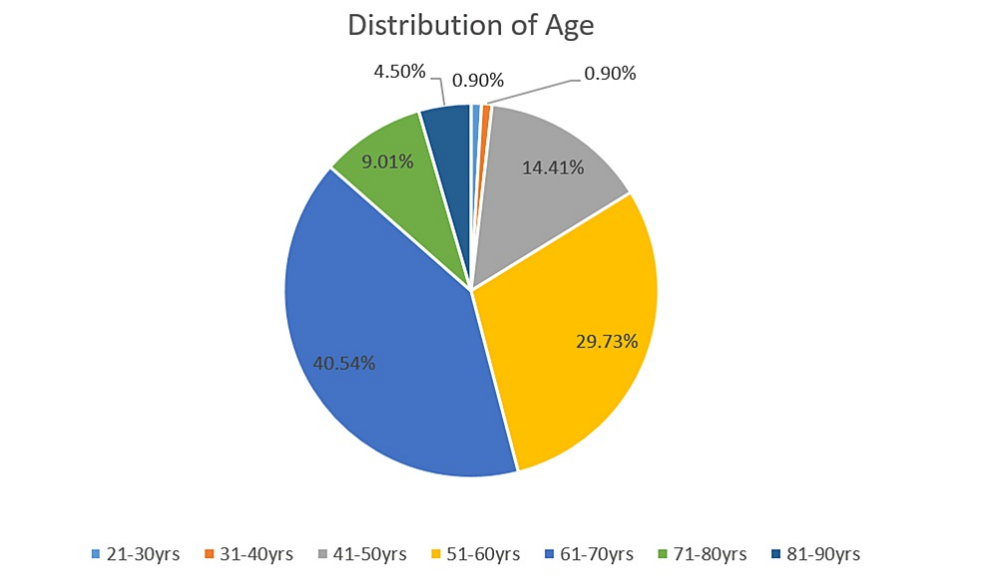
Distribution of age in OSCC patients. OSCC: Oral squamous cell carcinoma.

**Figure 3:**
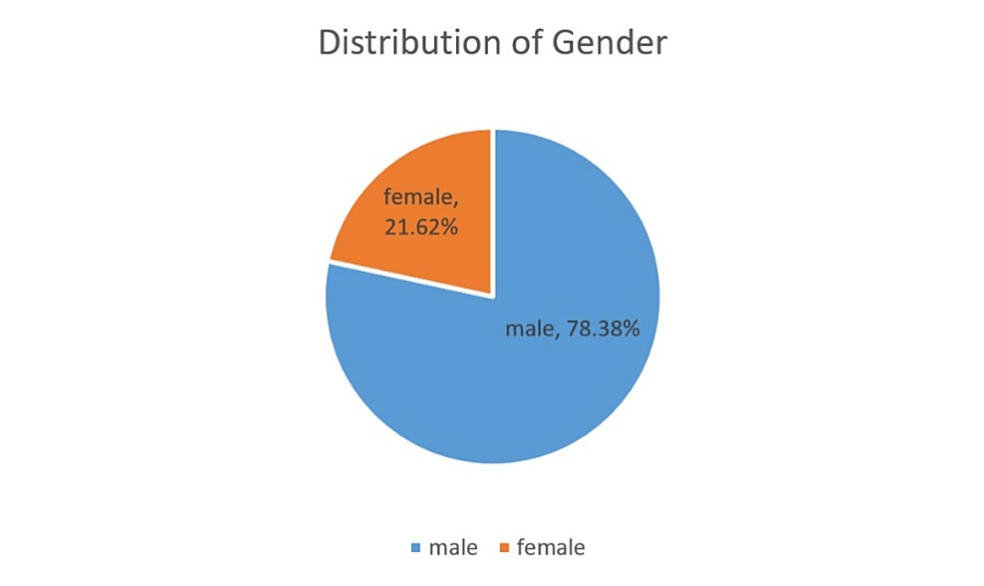
Distribution of gender of OSCC patients. OSCC: Oral squamous cell carcinoma.

The present study population includes 87 males (78.38%) and 24 females (21.62%) affected with OSCC. The participants were divided into seven age groups, ranging from 21-30 years to 81-90 years. The 21-30-year-old group and the 31-40-year-old age group had only one patient each (0.90%). There were 16 patients (14.41%) belonging to the 41-50 years' age group, 33 patients (29.73%) belonging to the 51-60 years' age group, 45 patients (4.50%) belonging to the 61-70 years' age group, 10 patients (9.01%) belonging to the 71-80 years' age group, and 5 patients (4.50%) belonging to 81-90 years' age group. Most of the study participants belonged to the 61-70 years' age group (40.54%), followed by the 51-60 years' age group (29.73%), while the 21-40 years' age group were the least affected. Figures 2, 3 represent the distribution of age and sex of the OSCC patients involved in the current study.

**Figure 4:**
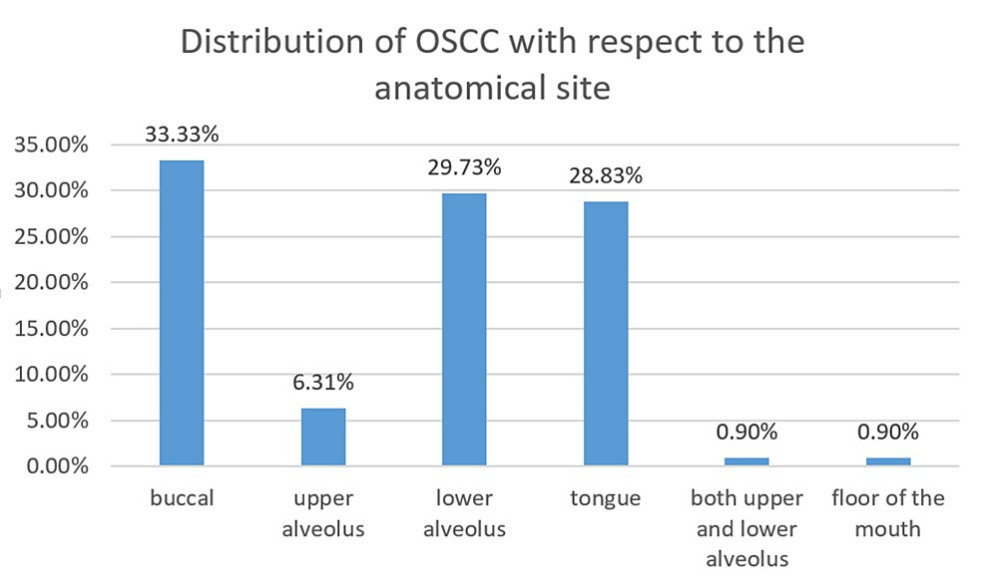
Distribution of OSCC in different anatomical sites. OSCC: Oral squamous cell carcinoma.

Based on the anatomical sites, OSCC was present in the buccal mucosa of 37 patients (33.33%), the lower alveolus of 33 patients (29.73%), the tongue of 32 patients (28.83%), and the upper alveolus of 7 patients (6.31%). Only one patient (0.90%) had OSCC involving both the upper and lower alveolus and the floor of the mouth. The buccal mucosa and the lower alveolus are the most commonly involved anatomical sites. Figure 4 depicts the anatomical distribution of OSCC patients.

The OSCC patients were classified based on the TNM staging including the extent of the tumor (T), nodal involvement (N), and metastasis (M) of the tumor. The extent of the tumor (T) was classified as T1 staging in six patients (5.41%), T2 staging in 33 patients (29.73%), T3 staging in 22 patients (19.82%), T4a staging in 45 patients (40.5%), and T4b staging in five patients (4.5%).

More than half of the study population including 65 patients (58.5%) did not have nodal involvement (N0). Regional lymph nodes cannot be evaluated (NX) in one patient (0.90%). Metastasis in a single ipsilateral lymph node with ≤ 3 cm in greatest dimension (N1) was present in 16 patients (14.41%). Metastasis in a single ipsilateral lymph node with >3 cm and ≤ 6 cm in greatest dimension (N2a) was present in four patients (3.60%), and metastasis in a multiple ipsilateral lymph nodes ≤ 6 cm in greatest dimension (N2b) was present in 14 patients (12.61%). Metastasis in a lymph node with >6 cm in greatest dimension (N3b) is present in 11 patients (9.91%).

**Figure 5:**
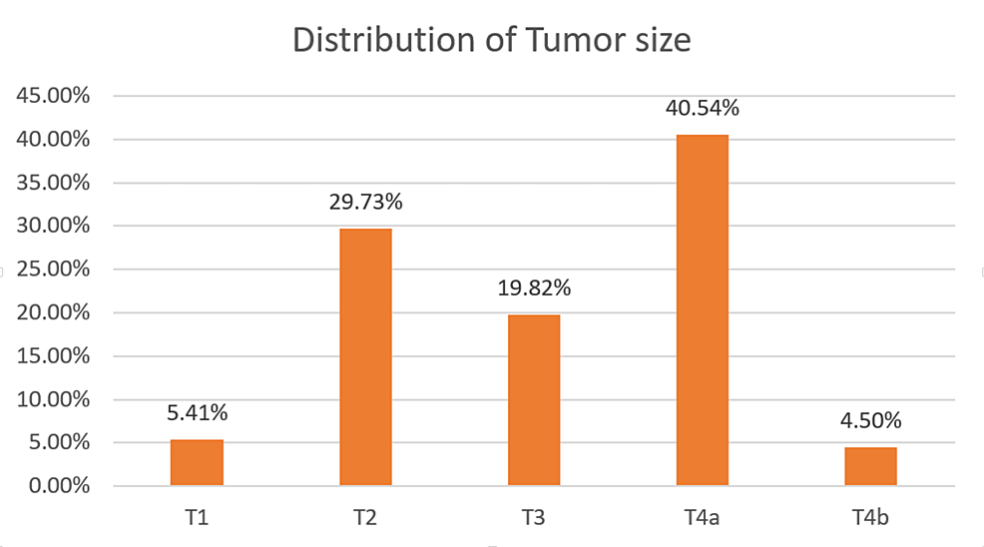
Distribution of OSCC in accordance with the tumor (T) staging. OSCC: Oral squamous cell carcinoma.

**Figure 6:**
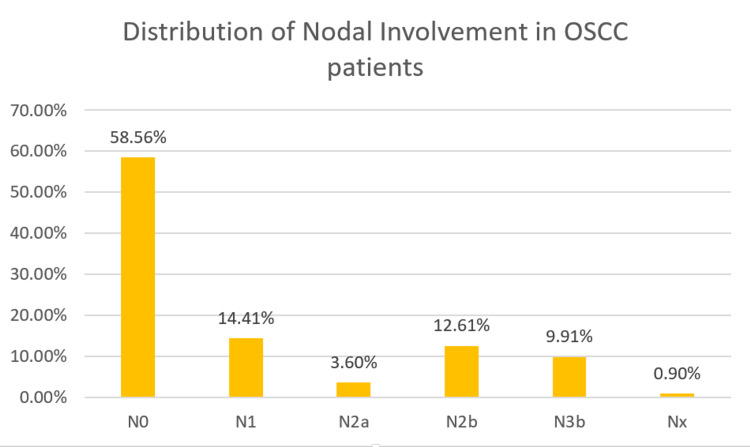
Distribution of OSCC with nodal involvement (N) staging. OSCC: Oral squamous cell carcinoma.

**Figure 7:**
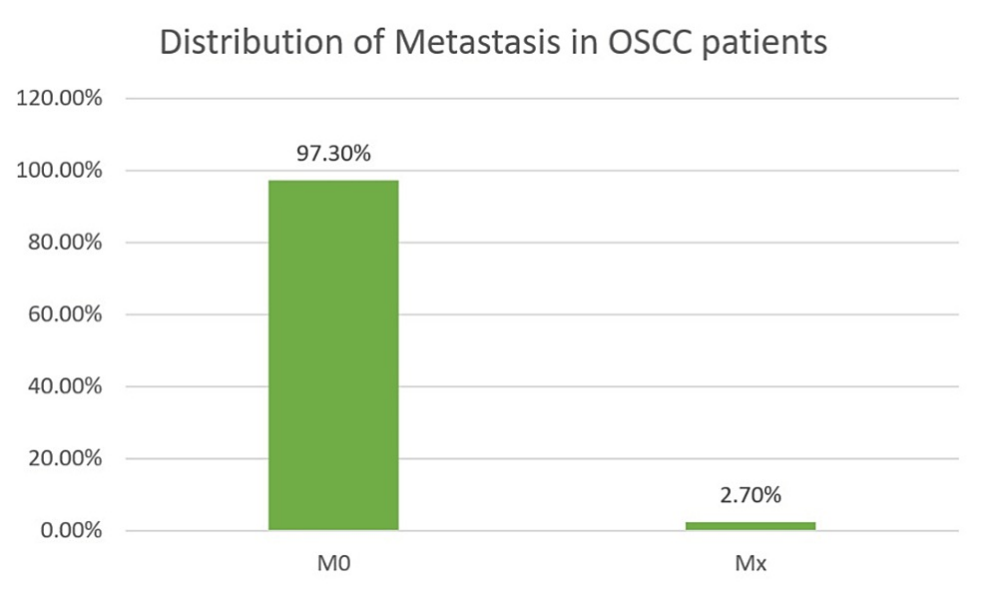
Distribution of metastasis (M).

Majority of the population (97.3%) did not have metastasis and were reported M0 in 108 patients. The distribution of the population based on the TNM staging is portrayed in Figures 5-7.

**Figure 8:**
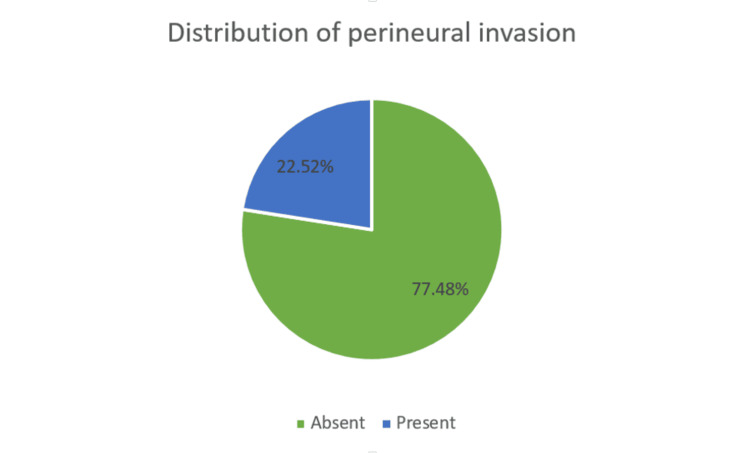
Distribution of perineural invasion of the OSCC patients. OSCC: Oral squamous cell carcinoma.

**Figure 9:**
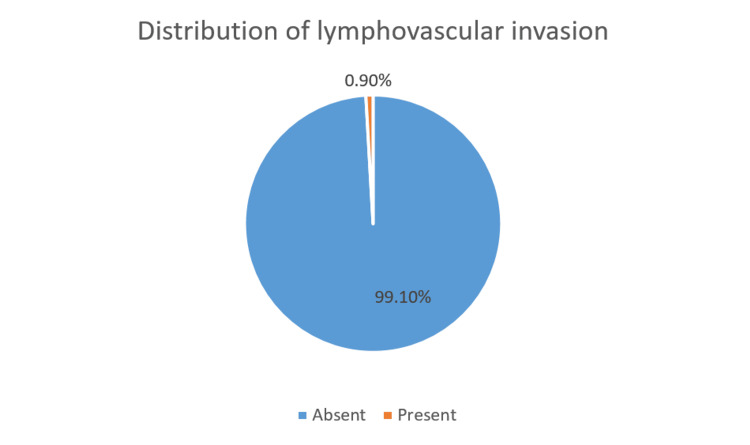
Distribution of lymphovascular invasion of OSCC patients. OSCC: Oral squamous cell carcinoma.

Majority of the study population including 86 patients (77.8%) did not have perineural involvement. Around 22.5% (25 patients) had perineural involvement. Lymphovascular involvement was not found in 110 patients (99.1%), and only one patient (0.9%) had lymphovascular involvement. Figures 8, 9 represent the percentage of perineural and lymphovascular invasion of the OSCC patients.

**Figure 10:**
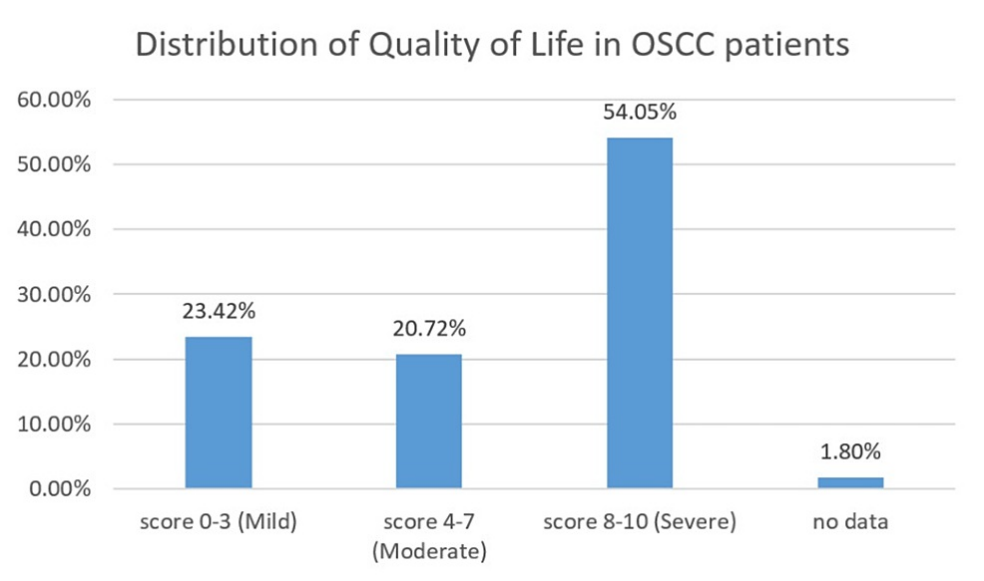
Distribution of quality of life in OSCC patients. OSCC: Oral squamous cell carcinoma.

Based on the discomfort in speech, swallowing, taste, mastication and pain, the modified OHIP score of the patients was assessed postoperatively. The quality of life score with a range of 8-10 was reported by 60 OSCC patients (54.05%), and the 4-7 score range was reported by 23 patients (20.72%). The least recorded score range of 0-3 was reported by 26 OSCC patients (23.4%). The quality of life of the OSCC patients is represented in Figure 10.

The correlation between gender and region, perineural invasion, lymphovascular invasion, quality of life, tumor staging as well as habit was analyzed through Pearson correlation. The Pearson correlation coefficient was represented by r. Gender has a very weak correlation with lymphovascular invasion (r = -0.05), a weak correlation with tumor staging (r=-0.208) and habit (r = -0.209), a moderate correlation with quality of life (r = 0.046), and a strong correlation with region (r = 0.073) and perineural invasion (-0.074). A statistical significance was observed between gender and tumor staging (p = 0.028) and habit (p = 0.027). The correlation of gender with other variables such as habit, anatomical site, tumor staging, perineural invasion, lymphovascular invasion, and oral health-related quality of life is depicted in Table 1.

**Table 1:** Association of gender with habit, quality of life, and OSCC *Statistically significant at p < 0.05, Pearson correlation. OSCC: Oral squamous cell carcinoma.

Gender		r	p
	Region	0.073	0.444
	Perineural invasion	-0.074	0.443
	Lymphovascular invasion	-0.05	0.602
	Quality of life	0.046	0.634
	Tumor staging	-0.208	0.028*
	Habit	-0.209	0.027*

## Discussion

The scientific study of the human population is referred to as demography. This kind of study helps in analyzing the distribution of population, changes happening within the population, and the various compositions of the group. This analysis provides a primitive understanding regarding certain diseases persisting within the population and the prevalence of the same [14]. Worldwide, Asian countries are found to have the highest prevalence of lip and oral cavity cancer, with males having a higher prevalence than females, as reported by the Global Cancer Observatory (GLOBOCAN) 2020. In India, oral cavity cancer is observed in the age group of 60-85 years, with a higher male than female population. The survey by the World Health Organization GLOBOCAN 2020 of India estimated that lip and oral cancer in the male population is 16.2% and 4.6% in the female population in India [15]. The male population was found to have a high dominance (78.3%) of OSCC compared with the female (21.62%) population in the current study. This was found to be in close approximation with a previous study, which stated that OSCC affected the male population at 77.9% and the female population at 22.1% [16]. Similarly, in Mexico, the prevalence of OSCC among men was 58.4%, and among women was 41.6% [17].

A notable gender bias exists, probably stemming from the greater willingness of males to adopt certain habits. The use of tobacco and betel nuts as stimulants makes males more vulnerable to oral cancer. In contrast, in India, the consumption of alcohol and tobacco has traditionally been discouraged among females. Nevertheless, this cultural norm is gradually diminishing, with females from various age groups and socioeconomic backgrounds increasingly embracing these habits [18]. The present study demonstrated a statistically significant correlation between habits and gender (p = 0.027). There was a 1.9-fold increased risk of oral cancer from smoking alone in men, while women faced a threefold increased risk. Higher daily cigarette usage and longer smoking periods further elevated the risk. After a decade, the risk of cancer returned to baseline levels for individuals who had abstained from the habit. One to two drinks per day was associated with a 1.7-fold elevated risk of malignancy among men; for heavy drinkers, the risk rose to about three times greater. An odds ratio (OR) of 35 was associated with cancer in those who drank more than four units of alcohol per day and smoked two packs of cigarettes or more [19,20].

The majority of cases of OSCC occur in older people, usually in their sixth or seventh decades of life. This is found to be in accordance with the current study, as the most affected age groups belong to the sixth and seventh decades of life. However, in contrast, a study done by Swetha Acharya et al. (2012) reported that 24.6% of OSCC patients were young Indian adults (>40 years). This increased incidence among the young population was attributed to early habitual exposure leading to greater susceptibility to OSCC [21]. The current study revealed that the most prone anatomical site for OSCC was the buccal mucosa region, followed by the lower alveolus region and the tongue. A study done by Akilesh et al. (2014) also stated that the commonest site of OSCC was the buccal mucosa in the North Indian population, which was in concordance with our study results [22]. 

A retrospective analysis by Garg et al. 2013 which was conducted to correlate the habit duration and risk levels of oral potentially malignant lesions revealed that patients with shorter habit duration (<5 years) had one third of high-risk lesions, whereas those with longer duration (>5 years) had two thirds of high-risk lesions [23]. Similarly, in the present study, comparison of tumor size staging with habit duration revealed that T4a patients were found to have a habit duration of more than five years. The staging is of prime importance for formatting the treatment plan and for the assessment of risk of recurrence and to ensure their quality of life [24]. In the current study, 22.52% of the population exhibited perineural invasion, and lymphovascular invasion was found to be 0.9%. Neurotropism is a key characteristic of OSCC, and the tongue exhibits abundant nerve distribution contributing to increased incidence rates of PNI approximately 28.3% [25,26]. It holds clinical significance, since it can be missed out by surgeons if not carefully assessed, thereby causing risk of recurrence [27]. Larsen et al. exhibited that perineural invasion was related significantly to the nodal involvement at the time of diagnosis of OSCC [28]. Lymphovascular invasion can be significantly used as a prognostic biomarker in case of OSCC since it has a significant correlation with the lymph nodal metastasis, where the route of metastasis is prevalently lymphogenic in nature [29]. Poor survival rate or compromised quality of life is indicated for patients with positive lymphovascular invasion [29]. This possibly explains the higher percentage of nodal involvement and severe compromise in oral health-related quality of life (score of 8 to 10-54.05%) of the individuals in the current study. Different treatment modality affects patients in different aspects, and it reflects on the posttreatment quality of life on the patient. Late-stage tumors typically require major surgical interventions along with adjuvant radiotherapy and/or chemotherapy attributing to difficulty with eating, swallowing, and tasting and reduced saliva production. Additionally, the psychological impacts can be attributed to anxiety, a lack of leisure time, and a sense of physical deterioration, adding to the overall difficulties of these patients [30]. Subsequently, the majority of the population underwent surgical dissection treatment for OSCC, and hence, their oral health-related quality of life is under the score of severe categories. Even though the study was presented with valuable results, some of the limitations are to be highlighted. Since it is a single-centered study, the data cannot be representative of other demographic regions. With the collected data being retrospective in nature, complete analysis of the study was impeded by incomplete information regarding the duration of the habit for certain patients and the individuals' quality of life.

## Conclusions

In this retrospective analysis, the buccal mucosa and the tongue are notably affected by oral squamous cell carcinoma in both sexes, and there is a strong association with harmful habits along with their duration and tumor staging, and gender is evident. Patient prognosis and quality of life are greatly impacted by the occurrence of lymphovascular and perineural invasions. It is imperative to set up public health initiatives aimed at educating people about oral cancer and expanding access to primary care, particularly for high-risk populations. 

## Appendices

**Table 2:** Modified OHIP questionnaire The overall score for the  Oral Health Impact Profile (OHIP)-7 is calculated by adding the scores from each of the five domains. The scores range from 0 to 10. Lower OHIP-7 levels imply improved well-being, while higher scores are associated with poorer oral health-related quality of life. The levels of severity are as follows: mild (0-3), moderate (4-7), and severe (8-10).

OHIP-7 Subscale and Items	Response	
1. Functional limitation (2 points)		
Difficulty in pronouncing words	Yes (1)	No (0)
Bad taste	Yes (1)	No (0)
2. Physical Pain (2 points)		
Had painful aching	Yes (2)	No (0)
3. Psychological discomfort (2 points)		
Feeling self-conscious	Yes (1)	No (0)
Feeling tense	Yes (1)	No (0)
4. Physical disability (2 points)		
Unsatisfactory diet	Yes (2)	No (0)
5. Handicap (2 points)		
Totally unable to function	Yes (2)	No (0)
